# Three Criteria of M-Type Spectrometers for Engineering

**DOI:** 10.3390/s25082439

**Published:** 2025-04-12

**Authors:** Zhaoqing Yang, Meng Xue, Hanming Guo

**Affiliations:** School of Optical-Electrical and Computer Engineering, University of Shanghai for Science and Technology, Shanghai 200093, China; 201310039@st.usst.edu.cn (Z.Y.); 201310037@st.usst.edu.cn (M.X.)

**Keywords:** Czerny–Turner spectrometers, luminous flux, aberration, Airy disk, optical resolution, detector resolution

## Abstract

Researchers frequently utilize the method of optical initial structure (MOIS) of Czerny–Turner (C–T) spectrometers for aberration-correction studies based on the coma-free equation. While effective, this method has limitations: small numerical apertures at slits (0.05–0.07) hinder weak signal detection; V or W-shaped variations in Airy disk across wavelengths; optical resolution depends on the radius of the collimating lens may not match detector resolution; and sequence patterns based on the spot diagrams cannot simulate the full width at half maximum (FWHM) under discrete sampling. To address these issues, using ray tracing and imaging equations, three criteria are proposed: luminous flux and aberration balance (LFAB), Airy disk variation at imaging points (ADVI), and optical-detector resolution matching (ORDR). A verification system with a 500–750 nm wavelength range and 0.4 nm resolution was designed. Results show that designing spectrometers based on these criteria increases the slit’s numerical aperture to 0.11 while controlling aberrations. After optimization, the tangential Airy disk size decreased by 28% with variations within 3 μm. Discrete sampling indicates FWHM pixel errors remain within 1/2 pixel of the theoretical value, and FWHM is at least 2.5 pixels, satisfying stricter sampling requirements beyond Nyquist. Optimization only involves adjusting the image plane by 0.017 mm axially, 0.879 mm off-axis, and 0.48° eccentricity. This research strengthens spectrometer design theory and improves practical applications.

## 1. Introduction

A spectrometer is an instrument for spectroscopic research and spectral analysis of substances and is widely used in clinical medicine, industrial detection, chemical analysis, and aerospace fields [1]. The classical Czerny–Turner (C–T) structure was initially introduced by M. Czerny and A. Turner [2]. It comprises two spherical mirrors and a plane grating situated along the parallel beam. Since 1960, due to the impact of aberrations in off-axis imaging systems, numerous studies have been devoted to aberration correction. Notably, the coma-free equation developed by Shafer has become a fundamental reference for the method of optical initial structure (MOIS).

To date, methods for aberration correction can be broadly categorized into two primary approaches. The first involves the incorporation of additional optical components [1,2,3,4,5], including the insertion of tilted lenses, the addition of cylindrical lenses or mirrors, and so forth. The second approach entails adhering to specific optical spacing configurations [6,7,8,9,10], such as fulfilling the first-order astigmatism-free conditions, employing grating divergent illumination, positioning the grating in proximity to the lens’s focal plane, and similar techniques. Undeniably, the above methods have all achieved good aberration elimination goals and made great contributions to the research on the aberrations of off-axis systems. However, it is not difficult to find that the MOIS and sequence evaluation systems referred to in the above studies still have certain limitations. On the one hand, in the research theories of aberration elimination based on the MOIS, the numerical aperture at the slit is typically in the range of 0.05 to 0.07. Nevertheless, as existing studies lack a theoretical criterion for balancing optical flux and aberration, designers have focused primarily on minimizing aberrations and achieving high-resolution imaging, leading to the selection of excessively small numerical apertures, which affects the detection of weak signals. Additionally, the inability to determine the nonlinear change trend of the Airy disk size at the imaging point leads to V or W-shaped fluctuations in the tangential RMS curve, with pronounced maxima and minima. This results in uneven aberration distribution across the full band, thereby affecting imaging quality. For example, in 2011 and 2012, Qingsheng Xue and Guo Xia confirmed that a wedge cylindrical lens and free-form cylindrical lens could be used for astigmatism correction. Although a resolution of 0.3 to 0.4 nm was achieved, the numerical aperture on the object side was required to be no greater than 0.05 [11,12]; In 2022, Siying Chen et al. utilized grating convergent illumination to correct astigmatism in the research of dual-excitation-wavelength aerosol spectral detection, and the numerical aperture at the slit was selected to be around 0.1, but there was no corresponding theoretical basis for the selection [13]; In 2009, Austin achieved broadband astigmatism compensation using divergent illumination. It not only required that the central wavelength from the focusing mirror to the detector be parallel to the central ray from the slit to the collimating mirror, but also that the F-number be approximately 8 [14], and the full-band RMS fluctuation range was from 4.5 μm to 12.5 μm. In 2013, Yan An derived the first-order astigmatism-free conditions for the central wavelength [7]. The finding indicated that the overall full-band RMS curve exhibited a V-shaped profile, leading to non-uniform aberrations across the entire band. In 2017, Qun Yuan and Guo Xia independently employed cylindrical lenses [3] and cylindrical mirrors [15] to correct astigmatism. However, the full-band RMS still displayed significant W and V-shaped variations. In the design result of eliminating astigmatism by matching a toroidal lens and a special filter by Ge in 2015, the RMS value in the high band was even four times that in the low band, with obvious maximum and minimum values [16]. In 2019, when Chen Wang and others were designing a 32-channel fluorescence detection spectrometer, they derived the first-order broadband distortion equation in order to better match the detector with the image plane. Although the NA was 0.1, the RMS in the meridional plane fluctuated as high as 22 microns in the working band, and the increase in the aperture angle affected the balance of aberrations [17]. In 2022, in the dual M-type spectrometer system with a shared linear detector designed by Pan Guo, when the working band was 280–460 nm, the middle and high bands were significantly lower than the low band, and the RMS curve variation value was about 30 microns. In the working band of 380–560 nm, the middle band was significantly lower than the low and high bands, and the variation value was about 25 microns [18]. Furthermore, the MOIS, due to its simple parameters, the initial optical resolution often mainly depends on the focal length of the collimating mirror. In the actual application process, it cannot be guaranteed that when the system parameters change before and after optimization, the reasonable matching relationship between the optical resolution and the detector resolution is maintained. It is highly likely to cause image plane waste or an inability to distinguish, lacking practical value. In 2014, Ting Ai Chen, while studying the central wavelength astigmatic elimination condition based on the concentric off-axis dual reflector system, suggested verifying the optical resolution using the Rayleigh criterion; however, the detector resolution was not taken into consideration [19]. In the 2016 study on the influence of a concave toroidal mirror on astigmatism, Dong posited that adjacent spectra could be distinguished as long as they did not overlap on the same pixel. However, he overlooked the fact that if the relative spectral intensities of adjacent pixels were similar, they would remain indistinguishable [20]. In 2022, Muddasir Naeem developed a geometric model based on design indicators, yet this model primarily concentrated on the effective image plane size of the detector, neglecting the pixel size and the actual discrete imaging process [21]. Similarly, in the same year, Wenjie Shi suggested using the Optical Transfer Function (OTF) to assess the resolution of the spectrometer [22]. Nevertheless, this approach only accounted for the slit size and failed to align with the parameters of the actual detector, lacking empirical validation. Theoretically, according to the Nyquist sampling theorem, the full width at half maximum (FWHM) of the imaging point at the image plane needs to correspond to at least two pixels. However, due to aberrations and assembly and adjustment effects, if the spectral resolution is judged only by this theorem, it is very likely to cause over-dense sampling and waste of pixels in some bands or insufficient sampling and affect the resolution in some bands. It is understood that there are currently no relevant detailed theoretical criteria.

On the other hand, it is not difficult to find that most studies adopt a spot diagram-based main sequence evaluation system. Due to the small slit, they often pay more attention to the imaging performance at the zero field of view and ignore the performance of the full field of view. At the same time, since this evaluation method cannot simulate the discrete sampling results of the detector on the image plane, the true spectral resolution cannot be obtained. Eventually, the optical performance can only be verified through actual measurement. For instance, Shucheng Li provided the Modulation Transfer Function (MTF) for the zero field of view in sequential mode and the spot diagram for balancing system aberrations by adjusting the grating position with a cylindrical lens in 2020 [23]. In 2022, Su Wu increased the numerical aperture on the object side by incorporating a hemisphere and directly assessed the 0.5 nm resolution index using the spot diagram and the RMS curve of the zero field of view [24]. Yingke Xie designed a near-infrared spectrometer based on an integrated scanning grating in 2023, which only presented the shape changes of the spot diagram before and after optimization through the sequence evaluation, while the actual spectral resolution could only be verified by the measured spectrum for its full width at half maximum [25]. To sum up, it is indispensable in the design process to optimize the sequence evaluation and find an evaluation method that can simulate the discrete sampling of the actual detector and accurately determine the spectral resolution.

It is worth mentioning that although the MOIS has a certain learning value, due to its simple parameters, a large amount of experience is often required in the design process, and the situation of relative position interference of each component is highly likely to occur during the optimization process [26]. We established the relevant model in 2024 and derived the anti-interference conditions of the M-type spectrometer, which further enhanced the reference value of the MOIS [26]. This article acknowledges the contributions of the MOIS in the design process of the C-T type spectrometer. At the same time, it is not difficult to find that in recent years, many researchers have also been focusing on the elimination of aberrations and related spectrometer design research. In order to further improve the completeness of the theoretical research on the spectrometer system, the following tasks have been undertaken. Firstly, three significant theoretical criteria have been established. Different from the previous research, we focus on solely pursuing the complete elimination of a single aberration. The spectrometer system is now defined using the F-number through the imaging relationship, thereby establishing the luminous flux and aberration balance criterion (LFAB). This approach allows for the selection of the numerical aperture at the slit to ensure adequate luminous flux while effectively controlling overall aberration. The Airy disk variation criterion at the imaging point (ADVI) for each wavelength is aimed at assessing the aberration variation across different wavelengths and fields of view, thereby providing a reference for the optimization process. Regarding the uneven distribution of imaging points for each wavelength on the image plane, a matching criterion between optical resolution and detector resolution (ORDR) has been derived. This criterion not only validates the rationality of the initial structural parameters but also provides a valuable reference for subsequent optimization efforts. Secondly, during the evaluation process of the spectrometer system, a non-sequential evaluation method has been incorporated. By elucidating the limitations of the spot diagram as the primary sequential evaluation metric, the significance of determining the actual spectral FWHM is highlighted. This involves considering the real luminous flux, slit size, and discrete sampling of the detector, while accounting for the effects of diffraction, to ensure enhanced accuracy in the system’s practical applications. The aforementioned research holds significant practical value, and it is noteworthy that no comparable systematic studies have been reported to date. Section 2 derives the aforementioned three theoretical criteria through the establishment of a geometric model in detail. In Section 3, building upon the preceding research, the anti-interference conditions within the method of initial engineering structure (MEIS) are integrated while ensuring the fulfillment of optical specifications, thereby obtaining a spectrometer structure that better aligns with practical application standards. A broadband, high-resolution spectrometer system is designed, featuring a wavelength range from 500 to 750 nm and a resolution of 0.4 nm, with a flowchart summarizing the design process. Section 4 provides a comparison of the design outcomes and evaluation metrics and compares the design results and evaluation indicators of the MOIS and the improved MOIS. The final section offers a corresponding summary and conclusion.

## 2. Theoretical Analysis

The C-T spectrometer is widely used in the field of high-resolution and wide-spectrum detection due to its simple structure and can avoid the stray light problem caused by secondary or multiple diffraction to a certain extent [7]. Meanwhile, the M-type structure is more widely used by designers because the coma and resolution are more stable within the working band compared to the cross-type structure. In order to meet the usage standards in different application fields, designers need to achieve design indicators of high received energy and high resolution in a wide spectral range, which often requires a more reasonable and complete theoretical system as well as stricter evaluation standards. The MOIS primarily emphasizes the optical resolution and the dimensions of the image plane [5,6]. Building upon this foundation, we will establish three critical viewpoints. Firstly, the F-number is introduced to refine the definition of the optical flux within the spectrometer system, thereby providing a condition for controlling spherical aberration. Secondly, by accounting for the impact of diffraction on the Airy disk imaging of various wavelengths on the image plane, we can set a benchmark for evaluating subsequent system optimizations. Lastly, we elucidate the cause of the non-uniform distribution of imaging points across different wavelengths on the image plane and derive the relationship formula linking optical resolution with detector resolution.

**Figure 1:**
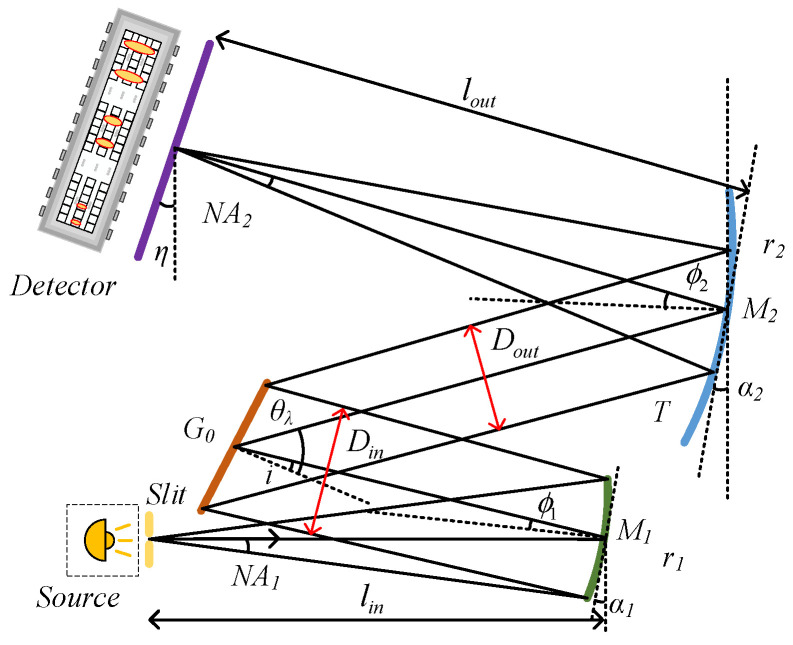
Schematic diagram of the Czerny–Turner spectrometer.

The classic M-type spectrometer structure is shown in Figure 1. The external light source passes through the slit at a certain divergence angle. The collimating mirror collimates the light beam and reflects it to the grating. The focusing mirror receives the diffracted light of the grating and focuses it to form an image on the detector. $r_{1}$, $r_{2}$, $\phi_{1}$, and $\phi_{2}$ are the curvature radii and off-axis angles of the collimating mirror $M_{1}$ and the focusing mirror $M_{2}$, respectively. The incident angle of the grating is denoted by i, and the diffraction angle is denoted by $\theta_{\lambda }$. $NA_{1}$ and $NA_{2}$ represent the numerical apertures of the slit entrance and the imaging point on the image plane, respectively. The actual deflection angle on the image plane is represented by η; $l_{in}$ and $l_{out}$ are defined as the front and rear focal lengths at the entrance and exit of the system, respectively. The grating serves as the system’s aperture stop. $D_{in}$ and $D_{out}$ are defined as the incident and diffracted light apertures of the grating, respectively.

In the design of spectrometer systems, the F-number is an important optical parameter, which can define the focusing ability of the light beam in the system. Usually, the entrance F-number and exit F-number of the spectrometer, respectively, describe the beam cone angle characteristics of the light as it enters the system from the light source and is transmitted from the system to the detector. $F_{in}$, which is the focal ratio when light passes through the entrance aperture or slit of the system, is defined as the ratio of the front focal length of $M_{1}$ to the diameter of the entrance beam. $F_{in}$ directly influences the luminous flux of the system, with its value determining the amount of light energy that the spectrometer can collect. When considering the impact of the off-axis angle, this relationship can be further refined.

(1) $$l_{in}=\frac{r_{1}}{2}\cos\phi_{1}$$

(2) $$D_{in}=r_{1}NA_{1}$$

(3) $$F_{in}=\frac{\cos\phi_{1}}{2NA_{1}}$$

$F_{out}$ defines the cone angle of the light beam projected from $M_{2}$ to the detector, and it can be expressed as the ratio of the rear focal length of $M_{2}$ to the diameter of the exit light beam. $F_{out}$ plays a crucial role in the spatial resolution of the system and the light energy utilization efficiency of the detector. A smaller exit F-number can focus the beam more tightly, thereby enhancing the signal strength and spectral resolution of the detector.

(4) $$l_{out}=\frac{r_{2}}{2}\cos\phi_{2}$$

(5) $$D_{out}=\frac{r_{1}NA_{1}\cos\theta_{\lambda }}{\cos i}$$

(6) $$F_{out}=\frac{r_{2}\cos\phi_{2}\cos i}{2r_{1}NA_{1}\cos\theta_{\lambda }}$$

Equation (7) can be obtained according to Equations (3) and (6),

(7) $$\left\{\begin{array}{l} F_{in}\propto \frac{1}{NA_{1}}\\ F_{out}\propto \frac{1}{NA_{1}} \end{array}\right.$$

which demonstrates that an increase in the numerical aperture at the slit can notably enhance the light flux at the entrance. Simultaneously, the focused spot received by the detector becomes more concentrated, thus improving the overall light energy reception efficiency. However, given that spherical aberration is closely associated with the aperture angle, the numerical aperture at the slit must be carefully controlled. As per the design criterion established by Lin Zhong et al. [23], the wave aberration produced by the spherical mirror should be maintained below one-quarter of the light wavelength.

Namely,

(8) D28r34≤λ4

r represents the curvature radius of the spherical mirror, D is the entrance pupil diameter of the spherical mirror, and λ is the working wavelength. In order to increase the luminous flux without overly introducing spherical aberration, which leads to increased aberration and reduced imaging quality. When considering the actual working wavelength, the off-axis angle of the collimating mirror, the curvature radius, and the numerical aperture at the slit, Criterion 1 LFAB should be followed, namely

(9) $$NA_{1}\leq 2^{\frac{5}{4}}{r_{1}}^{-\frac{1}{4}}\lambda^{\frac{1}{4}}\cos\phi_{1}$$

Equation (9) not only explains why the numerical aperture at the slit of the spectrometer is generally selected to be relatively small but also provides an upper limit value of the numerical aperture for increasing the luminous flux. The MOIS for calculating the resolution solely takes into account the FWHM corresponding to the geometric image width of the slit. However, it is known that high resolution often requires a small slit. When the slit is very small, for a system with small aberrations, the actual slit width is mainly determined by the diffraction image width. As the slit size gradually increases, so does the geometric image width. When the geometric image width equals the diffraction image width, the actual slit image width corresponds to the standard geometric image width. If the geometric image width exceeds the diffraction image width, the minimum resolvable wavelength difference of the spectrometer is dictated by the slit width. It is worth mentioning that although the RMS radius of the spectrometer in the tangential direction is often slightly larger than the Airy disk radius, the size of the Airy disk in each field of view can well help us judge the aberration size of each field of view and has a very good auxiliary effect on the realization of the resolution of the entire system. That is, according to the Lagrange invariant [25].

(10) $$nuy=n^{\prime }u^{\prime }y^{\prime }$$

(11) $$\frac{W_{S}^{\prime }}{W_{S}}=\frac{n\tan u}{n^{\prime }\tan u^{\prime }}=\frac{NA_{1}}{NA_{2}}$$

n is the refractive index of the object side, u is the aperture angle of the object side, y is the height of the object, and $n^{\prime }$, $u^{\prime }$, and $y^{\prime }$ correspond to the refractive index of the image side, the aperture angle, and the height of the image, respectively. $W_{s}$ is the width of the slit, and $W_{S}^{\prime }$ is the imaging width of the slit. According to the geometric imaging relationship: considering that the off-axis angles of the actual focusing mirror and collimating mirror are not large, the approximate calculation can be obtained.

(12) $$\frac{NA_{2}}{NA_{1}}=M_{G}\cdot \frac{f_{C}}{f_{F}},$$

$f_{C}$, $f_{F}$ is expressed as the focal length of $M_{1}$, $M_{2}$. The magnification factor $M_{G}$, which is the ratio of the diffraction light aperture of different wavelengths to the exit light aperture of collimating mirror, is related to the wavelength. d is the grating constant, m is the diffraction order.

(13) $$M_{G}=\frac{\cos\theta_{\lambda }}{\cos i}$$

(14) $$NA_{2}=\frac{\cos\theta_{\lambda }}{\cos i}\cdot \frac{f_{C}}{f_{F}}\cdot NA_{1}$$

(15) $$W_{S}^{\prime }=W_{S}\cdot \frac{\cos i}{\cos\theta_{\lambda }}\cdot \frac{f_{F}}{f_{C}}$$

(16) $$\Delta \lambda_{S-GEO}=W_{S}\cdot \frac{d\cos i}{mf_{C}}$$

Equation (16) takes into account the incident angle of the actual grating to determine the corresponding FWHM of the slit’s geometric imaging. Additionally, the imaging system exhibits a diffraction effect. Consequently, it is necessary to consider the impact of the slit diffraction width. The diffraction size of the slit imaging should adhere to Criterion 2 ADVI, which states that

(17) $$r_{Airy-disk}=\frac{0.61\lambda \cdot \cos i\cdot f_{F}}{\cos\theta_{\lambda }\cdot f_{C}\cdot NA_{1}}$$

It is evident that, when all other parameters are held constant and the wavelength progressively increases, the radius of the Airy disk exhibits a nonlinear increase. Given this nonlinear behavior, to ensure consistent resolution stability across the entire bandwidth, it is necessary to evaluate the correlation between the optical resolution of the full band and the detector resolution based on the changes in the Airy disk, rather than directly applying Equation (18) to determine the relationship between resolution and the operational bandwidth.

(18) $$\frac{\Delta \lambda }{L÷N}\geq 2$$

Δλ represents the resolution, N indicates the number of pixels, and L represents the working band. Equation (18) ignores the nonlinear influence of the spectral distribution, which is very likely to cause the actual situation of too few or excessive sampling of the detector. Ideally, if the spectral distribution is linear on the image plane, that is to say, the physical interval between different wavelengths is proportional to the wavelength difference, and C is a constant.

(19) $$\frac{\partial y}{\partial \lambda }=C$$

But due to the grating Equation (20)

(20) $$d\left(\sin\theta_{\lambda }+\sin i\right)=m\lambda$$

the spectral dispersion describes the physical interval corresponding to the unit wavelength interval on the image plane, considering the actual deflection angle of the image plane.

(21) $$\begin{array}{cc} \frac{\partial y}{\partial \lambda } & =f_{F}\cdot \frac{\partial \theta_{\lambda }}{\partial \lambda }\\ & =\frac{f_{F}\cdot m}{d\cos\theta_{\lambda }\cos\eta } \end{array}$$

The parameter $\frac{\partial \theta_{\lambda }}{\partial \lambda }$ denotes the rate of change of the diffraction angle with respect to the wavelength. This rate is not constant across all wavelengths, leading to varying distribution intervals of different wavelengths on the image plane. Specifically, as the wavelength increases, the rate of change of the diffraction angle accelerates, resulting in a relatively sparse spectral distribution. It is evident that when $\cos\theta_{\lambda }$ is sufficiently small, the non-linear distribution becomes more pronounced. To achieve a more uniform wavelength distribution, $\theta_{\lambda }$ should be minimized. In the past, to achieve high diffraction energy and an incident angle as close as possible to the auto-collimating incident angle, not only did nonlinearity increase, but there was also a heightened risk of component interference, rendering this approach unreasonable. Additionally, the deflection angle cosη of the detector should be minimized. This phenomenon, attributed to the diffraction characteristics of the grating, cannot be optimized or corrected subsequently. When used in conjunction with the detector, ensure

(22) $$\begin{array}{cc} \Delta \lambda & =\frac{np\cdot \partial \lambda }{\partial y}\\ & =\frac{dnp\cos\theta_{\lambda }\cos\eta }{f_{F}\cdot m} \end{array}$$

Subsequently, the Criterion 3 ORDR can be derived.

(23) $$np=\frac{W_{S}f_{F}\cos i}{f_{C}\cos\theta_{\lambda }\cos\varphi }$$

n is the number of pixels. Equation (23) integrates the pixel size, grating-related parameters, and the curvature radii of the collimating and focusing mirrors to account for the nonlinear effects of the grating. This approach allows for a more accurate determination of the matching relationship between optical and detector resolutions, thereby achieving a better balance between resolution and operational bandwidth. The above three criteria are derived based on the imaging relationship, with clear physical significance, and can provide a very good guiding role in the establishment of the initial structure of the spectrometer.

## 3. Modeling Establishment

The conventional spectrometer design frequently necessitates a certain level of design expertise. In summary, the design procedure involves initially estimating the grating line density based on the effective operating bandwidth and resolution requirements. To achieve high diffraction efficiency, the grating’s incidence angle should be minimized to approximate the auto-collimating incidence angle. The selection of the curvature radii for the collimating and focusing mirrors adheres to the basic imaging equation, while the deflection angle is determined using the coma-free equation as illustrated in Equation (24) [6]. This equation presumes that the off-axis angles of collimating mirror are minimal, typically not exceeding 10°.

(24) $$\frac{r_{2}}{r_{1}}=\sqrt{\frac{\sin\phi_{2}}{\sin\phi_{1}}{\left(\frac{\cos\theta }{\cos i}\right)}^{3}}$$

The above various initial parameters constitute the MOIS of the spectrometer. Under this initial structure, designers often need to optimize multiple times and use a large number of operands to achieve the optical design indicators and at the same time evaluate based on the system performance through the sequence mode [27]. Although based on the MOIS, by adding anti-interference conditions, the relative positions of its components are more reasonable to avoid mechanical interference, and the optimization is also relatively simple and suitable for beginners. However, from the perspective of practical application, we have found that there are still the following problems in the current theoretical research on the MOIS.

The MOIS overlooks the impact of the received luminous flux at the slit, specifically the size of numerical aperture’s, on the resulting aberrations.

The MOIS overlooks the variations in the Airy disks of imaging points across different wavelengths on the image plane, leading to a lack of a consistent standard for the subsequent optimization and control of the RMS of the imaging spot in the tangential plane. The fluctuating RMS curve suggests that the aberration performance over the entire wavelength range is insufficiently stable, potentially impacting the resolution.

When there is uneven imaging of each wavelength on the image plane of MOIS, the accurate correspondence between optical resolution and detector resolution cannot be established. As discussed in the Section 2, using the linear Equation (18) for calculation is prone to introduce a mismatch error between the optical resolution and the detector resolution.

The sequence evaluation adheres to the principle of ray tracing, with continuous sampling of the image plane, without accounting for the effects of actual slit diffraction. The spot diagram, which illustrates the separation degree of two adjacent wavelengths, fails to offer a more detailed assessment of resolution. In practical application, however, the detector employs discrete sampling, necessitating adherence to the Nyquist theorem. In order to ensure the accuracy of practical application, it is imperative to utilize the FWHM for a more accurate calibration of the resolution at each wavelength.

All of the above are problems that need to be solved urgently. This article is precisely committed to solving the above problems from practical applications. Based on the actual detector model, the size of the slit balances the relationship among the optical flux, resolution and working band, seeking a more reasonable, more practical, and more valuable spectrometer design system and verifying it. In this section, based on the theoretical system in Section 2, the spectrometer with a band range of 500–750 nm and a resolution of 0.4 nm broadband and high resolution is designed and evaluated. We previously established the anti-interference condition, as presented by Equation (25). $\alpha_{1}$, $\alpha_{2}$, and $\alpha_{3}$ represent the deflection angles of the collimating mirror, grating, and focusing mirror, respectively. $\Delta d_{1}$, $\Delta d_{2}$, and $\Delta d_{3}$ denote the axial distances between the center of the grating and slit, the center of the collimating mirror and focusing mirror, and the center of the image plane and grating, respectively. $l_{G_{0}M_{1}}$ and $l_{G_{0}M_{2}}$ represent the axial distances of the grating center from the centers of the collimating mirror and the focusing mirror. ${W_{F}}_{0}$ is the aperture size of the focusing mirror. $\beta_{F}$ is the angle between the lower-wavelength diffracted ray at the lower edge of the grating and the horizontal line. $\beta_{C}$ is the angle of the diffracted light of the central wavelength at the center of the grating relative to the horizontal line. γ is the angle between the reflected light of the low wavelength at the focusing mirror and the horizontal axis [26].

(25) $$\left\{\begin{array}{l} \frac{\tan2\alpha_{1}-NA_{1}\cos\alpha_{2}}{\tan2\alpha_{1}+NA_{1}}>\frac{2\Delta d_{1}}{r_{1}}\\ \tan2\alpha_{1}\cdot 2l_{G_{0}M_{1}}-D_{in}>D_{in}\cos\alpha_{2}-2\left(l_{G_{0}M_{2}}-\frac{W_{G}\sin\alpha_{2}}{2}\right)\tan\beta_{F}\\ l\tan\gamma >\frac{W_{F0}}{2}+\frac{W_{G}}{2}\cos\alpha_{2}-\tan\beta_{C}l_{G_{0}M_{2}}\\ l=l_{G_{0}M_{2}}-\frac{\tan\alpha_{3}W_{F0}}{2}-\frac{W_{G}}{2}\sin\alpha_{2} \end{array}\right.$$

The relevant design indicators are shown in Table 1 as follows.

**Figure 2:**
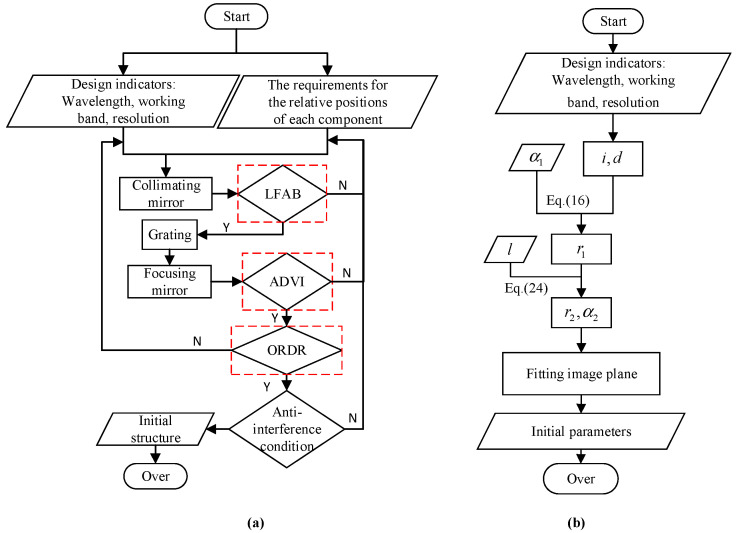
(**a**) Flowchart for improved MOIS; (**b**) flowchart for MOIS.

With achieving the optical indicators as the primary task, comprehensively considering the external dimensions of each component and the assembly and adjustment methods, we have formulated the relevant anti-interference margins. The initial values are set as $\Delta d_{1}=\Delta d_{2}=15\mathrm{mm}$, $Q_{G_{0}S}>4\mathrm{mm}$, $Q_{M_{1}M_{2}}>5\mathrm{mm}$, and $Q_{DG_{0}}>8\mathrm{mm}$. $Q_{G_{0}S}$ is expressed as the distance from the lower edge of the effective aperture of the grating to the vertical axis of the propagating ray. Suppose the vertical axis distance between the relative edges of $M_{1}$ and $M_{2}$ is $Q_{M_{1}M_{2}}$ and assume that the vertical axis of distance between the upper edge of the effective aperture of the grating and the focused ray is $Q_{DG_{0}}$. Combined with Equation (25), when $\alpha_{1}=6.5{}^{\circ}$, it is estimated that $r_{1}>95.763\mathrm{mm}$. By applying LFAB, specifically Equation (9), at this juncture, to prevent spherical aberration resulting from off-axis imaging, it is necessary that $NA_{1}\leq 0.113$. It is crucial not to indiscriminately increase the numerical aperture in pursuit of higher luminous flux. Similarly, setting the numerical aperture excessively low to mitigate off-axis aberration is also discouraged, as this can adversely impact the luminous flux. Considering that although a smaller slit size leads to higher resolution, it will cause more difficult assembly and adjustment as well as weakened signal intensity. At this time, if a 25 μm slit is matched, the grating constant is determined as d=1/1200mm and $f_{C}=52.083\mathrm{mm}$. Considering the influence of the secondary diffraction of the grating, from Equations (20) and (23), it can be obtained that i=7° and $\theta_{C}=38.912{}^{\circ}$. According to Equation (20), it can be obtained that

(26) $$l=\int_{\lambda_{1}}^{\lambda_{2}}\frac{f_{F}}{\cos\eta }\frac{m}{d\cos\theta_{\lambda }}d\lambda$$

where l represents the size of the detector’s imaging surface, $\lambda_{2}$ and $\lambda_{1}$ represent the maximum and minimum wavelengths within the effective band, respectively, and $f_{F}=74.1936\mathrm{mm}$. Based on $Q_{DG_{0}}>8\mathrm{mm}$ and $\Delta d_{3}<-20\mathrm{mm}$, $\alpha_{3}=-4{}^{\circ}$ can be obtained. Figure 2 illustrates the theoretical workflow in the practical spectrometer design system for improved MOIS and MOIS, with the red-marked sections indicating critical supplementary criteria. Meanwhile, for ease of understanding, Table 2 presents the comparison of the parameter selection criteria between the improved MOIS and the MOIS.

## 4. Design Results and Comparison

According to the above design process, the initial image plane position can be obtained based on the ray tracing, and the initial parameters of the positions of each component in detail are shown in Table 3.

In the past, the MOIS primarily aimed at eliminating coma aberration and enhancing optical resolution. Due to the limited complexity of parameters and the lack of consideration for the optimal relative positions of each element, designers often struggled to determine appropriate lengths for the parallel optical paths before and after the grating, resorting to setting arbitrary values initially. If significant interference arises in the initial structure during the optimization process, the deflection angles of the collimating or focusing mirrors are typically adjusted. The compact arrangement of components can lead to very tight spacing, making the MOIS less adaptable in practical applications. Table 4 gives the calculated parameters of the MOIS under the same design indicators. Because the actual effective image plane size is small and is not prone to interference with the grating, when considering that the actual overall size should not be too large, the grating constant in this working band is often selected as 1/1200. In order to ensure that the incident light direction does not coincide with the diffraction light direction, when the grating incident angle i=11°, the 500 nm diffraction light begins to coincide with the incident light of the grating, so i=9° is selected. In order to make full use of the imaging surface, it can be obtained from Equation (16) that $r_{1}\geq 52.083\;\mathrm{mm}$. Considering that the high-line-pair grating requires a larger incident angle for the same band range grating, in order to avoid obvious interference of the focusing mirror and collimating mirror caused by excessive grating deflection angle, $\phi_{1}=6.5{}^{\circ}$ is taken. The deflection angle of the focusing mirror is relatively small. According to Equations (24) and (26), it can be obtained that $r_{1}=-110.000\;\mathrm{mm}$, $r_{2}=-148.388\;\mathrm{mm}$, $\phi_{2}=22.382{}^{\circ}$. Meanwhile, the image plane position of the MOIS can be obtained by using the optical design software for fiber aiming.

**Figure 3:**
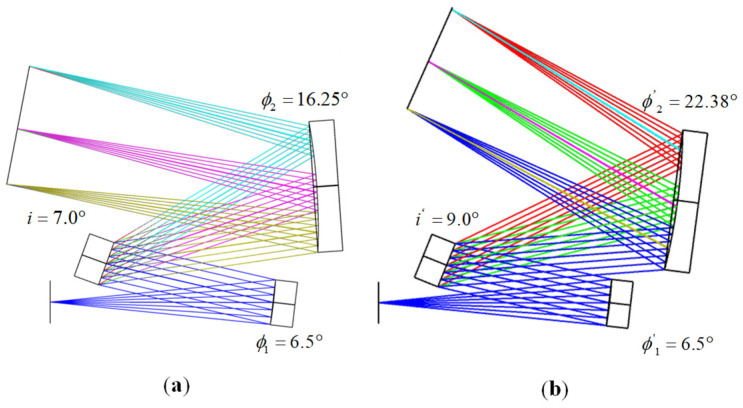
(**a**) Schematic diagram of improved MOIS; (**b**) schematic diagram of the MOIS.

As illustrated in Figure 3a represents the schematic diagram of the improved MOIS, which was calculated based on anti-interference conditions and integrated with the three primary criteria. From an analytical perspective, the relative positions of the various components effectively eliminate interference issues, thereby facilitating subsequent assembly and debugging processes. The overall optical path dimensions of this structure are 77 mm × 59 mm × 15 mm. In contrast, Figure 3b depicts the traditional MOIS centered around the coma-free equation. Simulation results confirm that the vertical distance between the collimating mirror and the focusing mirror in the MOIS is less than 2 mm, with an overall size of 78 mm × 77 mm × 25 mm. Meanwhile, in order to further compare the relative position changes of each component, we marked the working angles of the optical components in Figure 3a,b, respectively, for the readers’ understanding. Consequently, it is evident that the Figure 3a structure exhibits a significant advantage in terms of size compared to the Figure 3b structure and better aligns with the requirements for miniaturization design.

**Figure 4:**
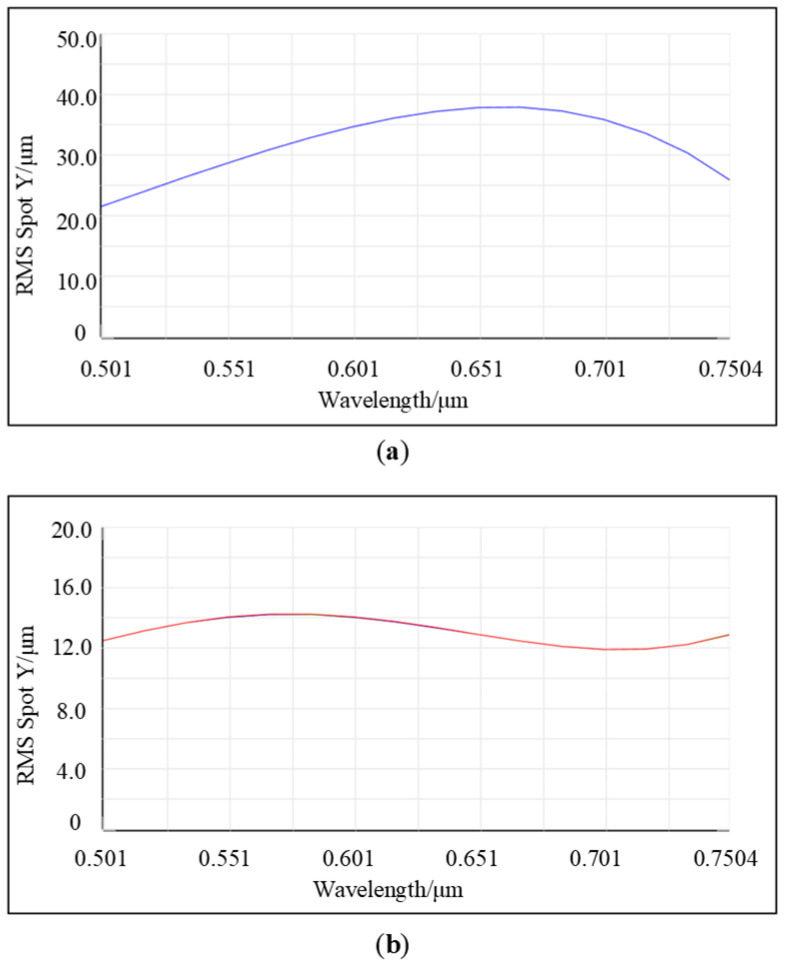
(**a**) Initial RMS curve for MOIS; (**b**) initial RMS curve for improved MOIS.

Figure 4 presents a comparative chart of the RMS curves in the tangential plane for two initial structures. Figure 4a represents the MOIS, which shows considerable RMS fluctuations with pronounced peak values that significantly deviate from the variation trend of the Airy disk at the imaging point. The maximum radius of the tangential Airy disk within the middle band can reach up to 37 μm, and the aberration distribution is uneven across the entire band. By contrast, Figure 4b illustrates the improved MOIS, where the RMS remains remarkably stable, with both maximum and minimum aberrations kept below 2 μm. This relatively low aberration in the initial structure greatly aids in the subsequent small-scale optimization of the system.

In Section 2, we have conducted a detailed analysis of the factors contributing to the non-uniform distribution of imaging points for each wavelength during the actual spectral imaging process. By employing Equations (17) and (23), we can ascertain the dimensions of the Airy disk for each wavelength under diffraction conditions, as well as the corresponding relationship between the optical resolution at each wavelength and the detector resolution, as presented in Table 5. On the premise of meeting the optical properties, not only the relative positions of each component are considered, but LFAB, ADVI, and ORDR are also combined to further ensure the accuracy of the design indicators and the initial structure, which also brings certain guiding value for the subsequent non-sequential evaluation simulation discrete sampling. Table 5 presents the number of pixels occupied by the FWHM of the imaging point of the improved initial structure and the calculated value of the Airy disk radius. If Equation (18) is adopted, the interval of the imaging points of adjacent wavelengths is consistently 3.3 throughout the entire wavelength band, which is larger in the low wavelength band and smaller in the high wavelength band, resulting in certain errors.

Taking the Airy disk radius in Table 5 as a reference, by keeping the other parameters unchanged and only optimizing the image plane position, an imaging point with smaller aberrations can be obtained, and the aberration distribution of each field of view of the slit is also more uniform. Table 6 illustrates the changes in the image plane position before and after optimization according to the criterion 2 ADVI. The slit is positive on the right and negative on the left. The thickness index given in Table 6 also follows the corresponding positive and negative sign rules. That is, the image center is to the left of the slit, so it is a negative sign. The variations in axial distance, vertical wheelbase, and angle adjustment are merely 0.017 mm, 0.879 mm, and 0.48°, respectively.

**Figure 5:**
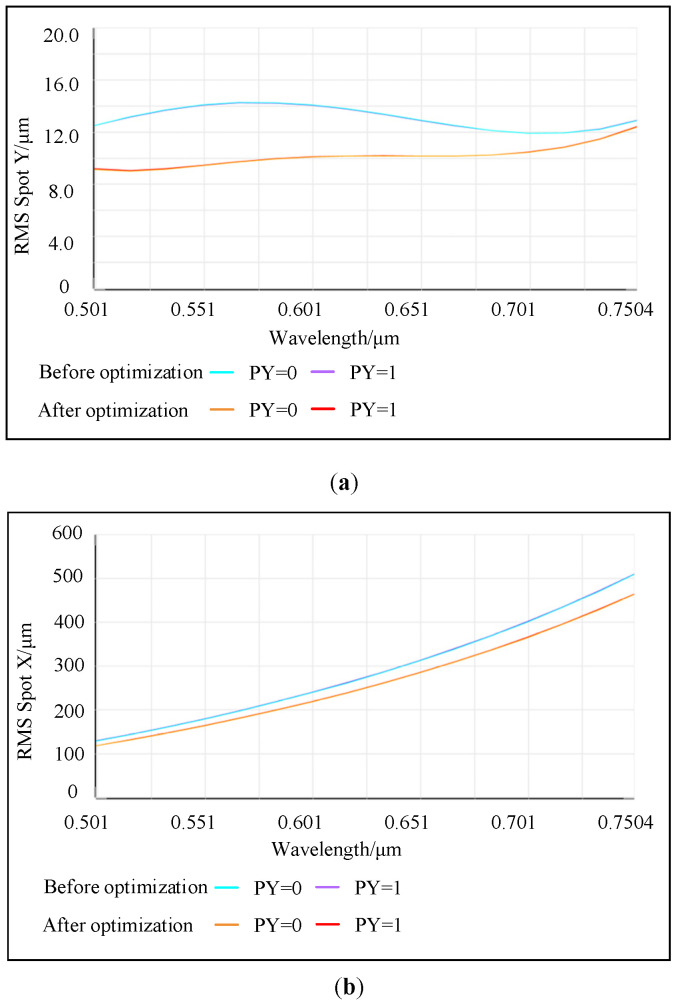
(**a**) The spot radius of the whole band on the tangential plane before and after optimization. (**b**) The spot radius of the whole band on the sagittal plane before and after optimization.

Figure 5 shows the comparison diagram of the RMS curves of the tangential and sagittal planes when a 25 μm slit size is selected. Figure 5a represents the RMS curve diagram of the tangential plane before and after optimization, and Figure 5b represents the RMS curve diagram of the sagittal plane before and after optimization, respectively. When PY = 0, it indicates a zero field of view, and when PY = 1, it represents a full field of view. The blue and orange curves illustrate the changes in the RMS values before and after optimization for the zero field-of-view scenario. Meanwhile, the purple and red curves show the changes in the RMS values before and after optimization for the full field-of-view scenario. It is not difficult to find through Figure 5a that the overall trend of the RMS in the meridian plane prior to optimization exhibits fluctuations. It neither aligns with the variation trend of the Airy disk radius for each wavelength across the full field of view nor corresponds to the distribution trend of wavelength imaging points on the detector. This is highly likely to result in uneven sampling by the actual detector, thereby impacting resolution. Following optimization, the overall decrease in RMS is as high as 28%, with a gentle trend and no significant extreme values, resembling the variation pattern of the Airy disk. This indicates that, despite the non-uniform distribution of wavelengths on the image plane, the trend in the pixel count at FWHM closely aligns with the RMS trend. This alignment facilitates discrete sampling by the actual detector and ensures stable resolution across the entire bandwidth. Additionally, the RMS uniformity across the zero field of view and the full field of view is exceptionally high, resulting in consistent image quality. It can be seen from Figure 5b that the maximum RMS of the sagittal plane before optimization was close to 500 μm, reduced to around 450 μm after optimization. The higher RMS in the sagittal plane compared to the tangential plane is mainly due to astigmatism. To control the sagittal spot, ensure there is no significant loss of energy detected by the detector. Both the ADVI and ORDR have provided positive guidance for the initial structure, enabling small-range image plane optimization to meet performance indicators.

**Figure 6:**
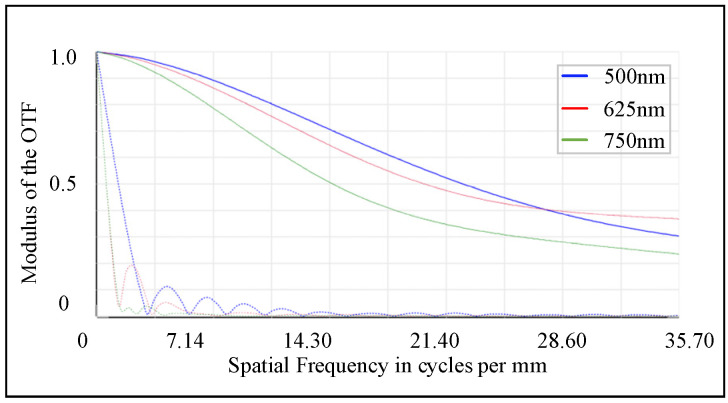
MTF diagram at the Nyquist frequency.

The modulation transfer function (MTF) curve sets the maximum value of the corresponding abscissa according to the Nyquist frequency. When the size of a single pixel is 14 μm, the corresponding Nyquist frequency is 35.7 lp/mm. Figure 6 shows the MTF curves at the Nyquist frequency, where different colors correspond to different wavelengths. It can be observed that the MTF values of each wavelength are generally high in the low and medium-frequency regions, while they decline relatively gently in the high-frequency region. Moreover, at the Nyquist frequency, the MTF values of all wavelengths are higher than 0.2, which is consistent with the frequency response characteristics of the detector. At the same time, the curves show continuity throughout the frequency range, and there is no sudden cut-off at certain frequencies. This feature further proves the good quality of the imaging system.

Subsequently, we will conduct a thorough evaluation, comparison, and analysis of the sequence mode spot diagram relative to the non-sequence mode incoherent irradiance schematic. In sequence evaluation, all light propagation occurs on the optical surfaces within a specific local coordinate system. In non-sequential evaluation, ray tracing involves scenarios where the target surface for the traced ray is not predetermined. By configuring the detector’s pixel size and the number of discrete samples, a more accurate representation of image formation on the image plane can be achieved. By setting the slit size and the numerical aperture at the slit, and tracing 10^8^ rays, we can simulate the energy reception on the image plane using a detector with 2048 pixels, each having a size of 14 μm.

**Figure 7:**
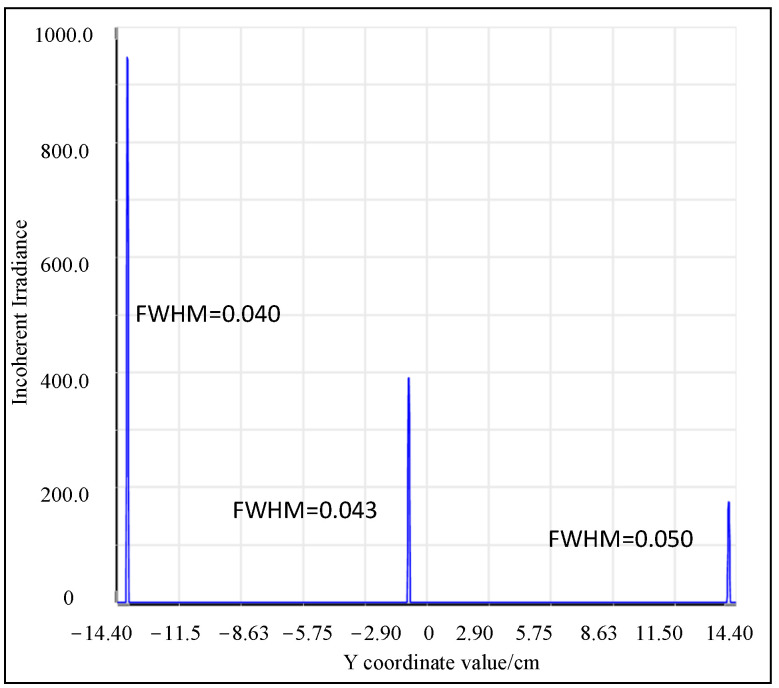
Schematic diagram of incoherent irradiance.

Figure 7 illustrates the incoherent irradiance, which is used to determine the FWHM for each wavelength, thus providing the precise and optimal resolution. Table 7 presents the image plane intervals corresponding to the FWHM of each wavelength, along with their respective FWHM values. It is evident that the overall design band satisfies the resolution index requirements and the resolution variation of the full band is less than 0.006 nm. However, considering the minor impact of actual aberrations, this discrepancy is primarily due to the grating equation causing the diffraction angle to vary nonlinearly with wavelength, leading to a slightly different theoretical FWHM in the full-wave band compared to the actual results. According to the data comparison, the pixel error of the FWHM at the image plane for each wavelength imaging point remains within 0.5 pixels. While theoretically, the detection signal can be fully reconstructed in accordance with the Nyquist sampling theorem, practical considerations necessitate accounting for additional processing and assembly errors, which are empirically estimated to be around 10%. Consequently, it is advisable for designers to allocate a specific margin during the design phase. Thus, we recommend that in the initial structural calculations, n≥2.5, this inequality should serve as a valuable reference for optimizing the spectrometer design to balance resolution and operational bandwidth.

**Figure 8:**
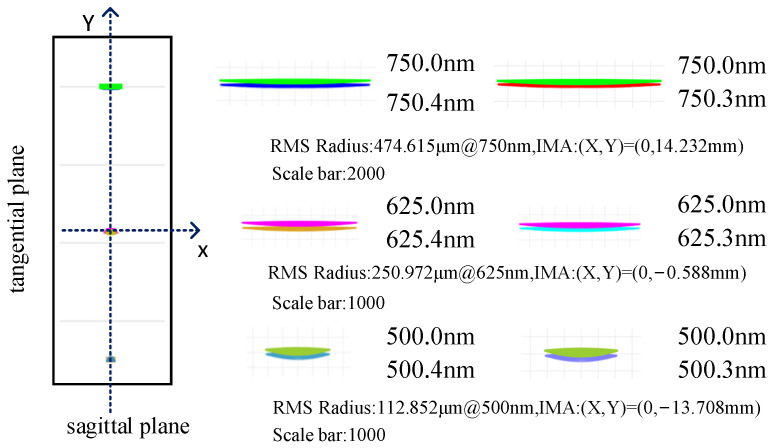
Full-band spot diagram corresponding to the resolutions of 0.4 nm and 0.3 nm.

The spot diagram, as a prevalent method for evaluating sequence, illustrates the separation degree of adjacent wavelengths on the image plane. When considering actual resolution, it is not solely dependent on the relative distance between adjacent wavelengths on the image plane but also necessitates taking into account the size of the imaging point and the resolution capability of the detector. Figure 8 illustrates the separation of image points when the intervals between two adjacent wavelengths are 0.4 nm and 0.3 nm, respectively, within the working wavelength band. Each color corresponds to a different wavelength. In Figure 8, we have illustrated the coordinate axes using dotted lines, with the center of the image plane serving as the origin. Here, X denotes the sagittal direction, while Y indicates the tangential direction. The position coordinates of the image plane, RMS radius values, and the corresponding magnification coefficients for a single view at wavelengths of 750 nm, 625 nm, and 500 nm are clearly marked. It is important to note that, due to the effects of astigmatism, the RMS radius corresponds specifically to the sagittal direction. From the above analysis, the system achieves a resolution of 0.4 nm after optimization. While the spot diagram shows that adjacent wavelengths at 0.4 nm and 0.3 nm intervals are visibly separated, the final half-width analysis indicates the design does not reach 0.3 nm. Thus, judging spectral resolution solely by the spot diagram in the design process is unreasonable.

**Figure 9:**
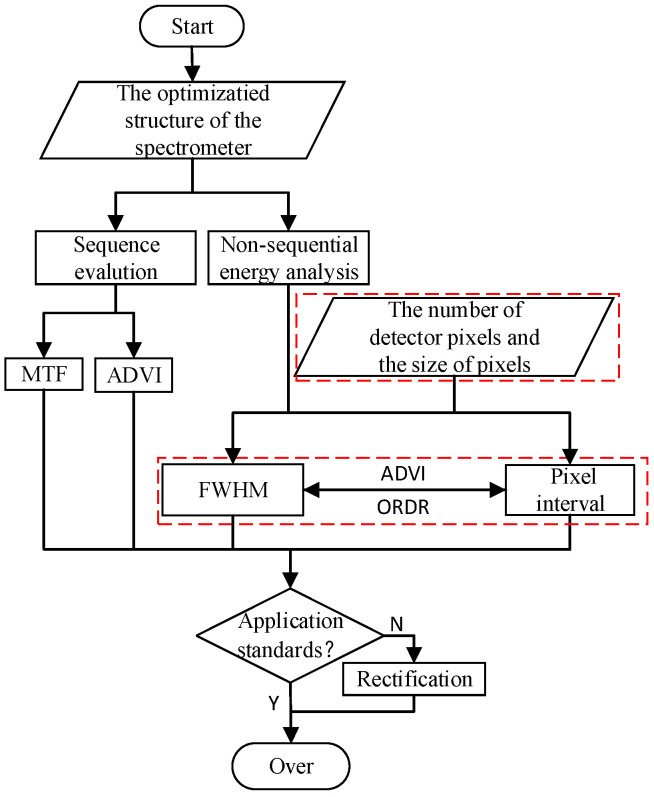
Flowchart for evaluating the spectrometer structure.

To sum up, we summarize and obtain the flow chart of the improved evaluation system as shown in Figure 9. As is known to all, an excellent spectrometer system must have certain practical value. Both the MOIS and the sequence evaluation method have certain defects in the actual application process. It is indispensable to study more reasonable and more valuable criteria and evaluation methods in the field of spectrometer design and application.

## 5. Conclusions

The main research idea of this article emphasizes taking the realization of optical properties as the top priority, further enhancing the completeness of the M-type spectrometer system theory, and providing certain reference values for practical applications at the same time. It is undeniable that the MOIS based on the coma-free equation provides certain learning ideas for the design of spectrometers. However, according to the analysis, the MOIS is more suitable for experienced designers due to its simple parameters. It is known that in recent years, many researchers have still been focusing on aberration elimination and spectrometer design research. In 2024, we introduced anti-interference conditions based on our previous work. Ensuring the optimal placement of each component, subsequent optimization adjustments were minimal and more accessible to beginners. Given the existing limitations in the theoretical research of the MOIS, we established three critical theoretical criteria, providing them with clear physical interpretations. These criteria encompass the luminous flux and aberration balance criterion (LFAB), the Airy disk variation criterion at the imaging point (ADVI) to establish a benchmark for subsequent system optimization evaluations, and the optical resolution and detector resolution matching criterion (ORDR). Additionally, a broadband, high-resolution spectrometer system has been designed, featuring a wavelength range of 500–750 nm and a resolution of 0.4 nm. The results show that based on the above criteria, a reasonable numerical aperture of 0.11 is selected. the improved initial structure based on the above criteria not only has a smaller size and more reasonable phase positions of each component but also only needs to change the image plane position before and after optimization. The axial distance, off-axis, and eccentricity changes are only 0.017 mm, 0.879 mm, and 0.48°, respectively. These changes reduce the tangential Airy disk size by 28% with a slow upward trend and the difference between the maximum and the minimum is less than 3 μm. The error of the actual FWHM in the non-sequential evaluation and verification does not exceed 1/2 pixel, which can fully ensure the realization of each optical index. Considering the influence of aberration comprehensively, we suggest that the FWHM of the image point should correspond to at least 2.5 pixels. This study offers valuable insights for the development of high-throughput, high-resolution, and wide-spectrum spectrometers in future research.

## Figures and Tables

**Table 1:** Design specifications of improved M-type spectrometer.

Parameter	Value
Spectral range	500~750 nm
Resolution	0.4 nm
Effective image plane size of detector	28.672 × 0.896 mm
External dimension of detector	48 × 12.7 × 6 mm
Pixel size	14 μm
Slit size	25 μm

**Table 2:** Comparison of relevant parameters between the improved MOIS and MOIS.

Parameter	Improved MOIS	MOIS
NA	LFAB: >0.1	Default: 0.05~0.07
RMS—tangential	ADVI: no obvious extreme values.	V or W-shaped variations
Resolution	ORDR: optical & detector	optical
Evaluation	non-sequential	sequential
	discrete sampling of the image plane	continuous sampling of the image plane

**Table 3:** Parameters and positions of each component before optimization of the improved MOIS.

Parameter		Value
$M_{1}$	$r_{1}$ (mm)	−104.1667
	Decenter Y (mm)	0
	Tilt X (°)	6.5000
	Thickness (mm)	51.798
	$W_{C}$ (mm)	10.542
$G_{0}$	d (mm)	1/1200
	Decenter Y (mm)	8.957
	Tilt X (°)	20.000
	Thickness (mm)	13.000
	$W_{G_{0}}$ (mm)	10.470
$M_{2}$	$r_{2}$ (mm)	−148.3872
	Decenter Y (mm)	26.914
	Tilt X (°)	−4.000
	Thickness (mm)	61.798
	$W_{F}$ (mm)	30.5456 × 10.58
Image	Decenter Y (mm)	40.278
	Tilt X (°)	10.52
	Thickness (mm)	−7.419

**Table 4:** Parameters of the MOIS.

Parameter		Value
$M_{1}$	$r_{1}$ (mm)	−110.000
	Decenter Y (mm)	0
	Tilt X (°)	6.5000
	Thickness (mm)	55.020
$G_{0}$	d (mm)	1/1200
	Decenter Y (mm)	8.957
	Tilt X (°)	22.000
	Thickness (mm)	16.202
$M_{2}$	$r_{2}$ (mm)	−148.388
	Decenter Y (mm)	24.457
	Tilt X (°)	7.400
	Thickness (mm)	71.202
Image	Decenter Y (mm)	58.457
	Tilt X (°)	24.000
	Thickness (mm)	12.002

**Table 5:** The relevant criterion results of the M-type spectrometer.

Wavelength (nm)	The Number of Occupied Pixel	The Radius of Airy Disk (μm)
500	2.9	4.905
625	3.3	6.921
750	3.9	10.288

**Table 6:** Parameters of the image plane before and after optimization of the M-type spectrometer.

Parameters	Initial Value	Optimized Value
Thickness (mm)	−7.419	−7.402
Decenter Y (mm)	40.278	41.157
Tilt X (°)	10.520	11.000

**Table 7:** The relevant criterion results of the M-type spectrometer after optimization.

Wavelength (nm)	FWHM (mm)	The Number of Occupied Pixel	Resolution (nm)
500	0.040	2.85	0.391
625	0.043	3.07	0.386
750	0.050	3.57	0.385

## Data Availability

The data that support the findings of this study are available from the authors upon reasonable request (see Author Contributions for specific data sets).
